# Medical Wards of the Mercy Hospital

**Published:** 1860-02

**Authors:** N. S. Davis


### CLINICAL REPORTS.

Medical Wards of the Mercy Hospital. Service of Prof. N. S. Davis.

Nov. 8th, 1859. Male Ward No. 2. On entering the ward, Dr. Davis remarked to the class, that he should restrict their attention during the present clinic hour to two cases which were worthy of a very careful examination.

The first is a man, native of Ireland, aged about 45 years, who came a few days since from the southern part of Wisconsin, with the hope of obtaining relief from an abdominal dropsy. He observed that the patient was considerably emaciated; the lips thin, though not pale; the conjunctiva of the eye slightly yellow; the skin dry, but of natural temperature; the tongue natural; the bowels inactive or constipated; digestion impaired, producing much gaseous eruetations after meals; sense of fullness and oppression in the epigastric and hypochondriac regions; frequent sharp pains, with some tenderness in the region of the right ureter; urine scanty and high colored; pulse 85 per minute, and moderately full; and the whole abdomen much distended with a fluid, as shown by percussion and succussion. After giving the class an opportunity to examine the abdomen individually, he remarked that in former times *dropsy* was regarded as a disease *per se.* And hence, nearly all the older writers will be found to treat of two varieties, viz: Active and passive, or sthenic and asthenic dropsies. But modern researches have clearly shown all the forms of dropsy to be the result or consequence of some preceding pathological condition, often located in some part remote from the seat of the dropsical effusion. Hence the accumulation of water or serum in the abdomen of this patient, as in all others, is to be regarded as a mere symptom, as much so as a cough, a hot skin, or a pain in the head. Whenever, therefore, a patient is presented to you with dropsical accumulations, whether in the form of anasarca, ascites, or hydrathorax; the first question which should occupy your minds is, what is the cause, or special pathological condition, which has given rise to this effusion? For on a proper solution of this will depend all rational treatment. Your ability to arrive at clear and accurate conclusions in relation to any particular case, will be greatly enhanced by keeping in mind the following general considerations, viz: First, all dropsies arise immediately from, either such an alteration in the relative proportion of the proximate elements of the whole mass of the blood as to leave the *watery* element in excess, or from direct mechanical obstruction of the blood vessels connected with the seat of the dropsy.

Second, in all cases in which the dropsy arises from an alteration of the whole mass of the blood, it first shows itself in the areolar tissues, and most prominently in the parts that are most dependent or most remote from the heart; and hence is termed œdema or anasarca. But by continuance it may, not only invade all the areolar tissues, but also one or all the serous sacs of the body, as the pleura, peritoneum, pericardium, etc., and is hence often called *general* dropsy. On the other hand, in all those cases where mechanical obstruction to the circulation is the immediate cause, the dropsical accumulation is limited to the part with which the obstructed vessels are connected; and hence it is called circumscribed or *local* dropsy.

The pathological conditions capable of giving rise to what he had defined as general dropsy, are numerous. Those of most practical importance are; First, albuminuria, or such an alteration of the function of the kidneys, as causes a portion of the albumen of the blood to escape with the urine, and thereby leaves the watery element in excess; second, protracted organic disease of the heart; third, protracted interruption of the menses, or long continued action of malaria; either of which are capable of rendering the individual so anemic, or, in other words, of producing such a diminution of the red corpuscles of the blood, as to leave the water greatly in excess.

The pathological conditions capable of giving rise to local dropsy, are two, viz: Such a mechanical pressure on the trunks of the blood vessels as to cause their capillary extremities to become habitually over distended, until the watery part permeates the coats of the vessels, and infiltrates the interstitial spaces, or is effused upon a membranous surface; and direct inflammation of the texture or membrane from which the effusion takes place.

As both these conditions are restricted in their action to particular vessels or tissues, so the resulting dropsical effusion must be limited to corresponding parts, and therefore necessarily local. Applying these general observations to the case before us, the question immediately arises, is it one of general or local dropsy? On examination we find it belonging to the latter class, for the accumulation of fluid is limited exclusively to the peritoneal sac. This determined, we advance directly to another question, namely, whether the accumulation is the result of pressure upon some of the vessels connected with the abdominal cavity, or of inflammation of the peritoneal membrane itself?

Peritonitis, acute or chronic, would be accompanied by more or less febrile movement; a sharp and quick pulse; tenseness of the abdominal walls, with decided tenderness; and a rapid wasting of flesh and strength. The patient before us has none of these symptoms, except the emaciation which has been produced very slowly, and is only of moderate amount, and a very limited degree of tenderness in the right iliac region. From the absence of all the more important symptoms of peritoneal inflammation, we must look for some one of those causes which mechanically obstruct the abdominal circulation, such as alterations in the size and texture of the liver, spleen, and mesenteric glands. To detect the existence of these, we must rely principally upon palpation or touch aided by percussion. Placing the patient in a recumbent position, with the abdominal muscles relaxed, the lecturer proceeded to make a careful physical exploration of the abdomen. No solid body could be felt in the abdominal cavity, and the accumulation of water rendered it everywhere dull on percussion, except directly over the course of the transverse colon and stomach, where the tympanitic or intestinal resonance was well marked. He pointed out the latter fact as one of much diagnostic value in such cases. The natural position of the transverse colon, being immediately beneath the lower edge of the liver and spleen; it must necessarily be carried downward whenever either or both of these organs become enlarged by disease; thereby extending the hepatic or splenitic dullness below the lower margin of the ribs.

But in this patient, the resonance of the colon not only exists up to the convex edge of the ribs, but extends, on the right side, more than an inch above the lower margin of the hypochondriac region. This clearly shows that the liver is not enlarged. Neither do the physical signs afford evidence of enlargement of any of the abdominal viscera. But there is another disease of the liver besides enlargement, that so far obstructs the portal circulation, as to make ascites one of its most constant accompaniments. He alluded to cirrhosis or hob-nail liver; a disease which induces such a degree of contraction and atrophy of that organ, as to obstruct the flow of blood through the hepatic capillaries of the vena portæ, thereby causing the trunk of that vessel and its intestinal capillaries to be over-distended, until the serous or watery portion of the contained blood becomes effused into the sac of the peritoneum.

Extending the examination by percussion over the whole of the right side of the chest, it was found that the hepatic dullness covered a much less space than natural, being less than two inches in vertical diameter. There was also some tenderness over the region of the liver as shown by the complaint of the patient during the act of percussion. If we take these facts in connection with the slow development of the disease; the frequent evidence of hepatic derangement during its early progress; the constantly disordered digestion; the torpid state of the bowels; the scanty and high colored condition of the urine; *followed* by the gradually increasing dropsical effusion into the cavity of the abdomen, there can be scarcely a doubt but the case is one of genuine cirrhosis; advanced to that stage which is characterized by decided atrophy and contraction of the liver, with functional disorder of the whole digestive aparatus, and serous effusion.

The origin and nature of the disease termed Cirrhosis, is involved in obscurity. It has been supposed to originate in a low grade of inflammation in the fibrous tissue that enters the liver surrounding the hepatic vessels, and extending with them around each separate *lobule*; and that the subsequent atrophy and contraction is the result of obstruction to the nutrient arteries and contraction of the fibrous texture. This view is countenanced by the fact that in most, if not all of the cases, there is in the early stage more or less pain and tenderness in the hepatic region, with some fever. Such was the case with this patient in the early stage, and you have seen that the tenderness still continues in the case before us. The disease occurs much the most frequently in those who are addicted to the use of alcoholic beverages.

When the disease has continued, as in the case before us, until the size of the organ is much diminished, and the portal circulation sufficiently obstructed to cause ascites, the prognosis is very unfavorable. The dropsical accumulation may be retarded by diuretics, or it may be temporarily removed by paracentisis abdominis, but the loss of flesh and strength continues to increase gradually, until at length a fatal degree of exhaustion supervenes. The Doctor remarked that he should prescribe for the present patient a powder of hydrarg. cum. creta 2 grs., and pulv. doveri 3 grs., to be given before each meal; and an infusion of juniper berries, uva ursi, and digitalis leaves, in doses of half a wine-glassful every four hours. After the alterative powder had been taken for two days, it should be followed by a laxative sufficient to move the bowels. If the diuretics fail to lessen the amount of effusion, and the abdomen becomes so much distended as to interfere with respiration, tapping must be resorted to for temporary relief. He alluded to some cases in which a large amount of fluid had been evacuated four or five times through the canula of the trocar, before the period of fatal exhaustion was reached.

Case 2d. *Chronic Conjunctivitis.* The second case to which the attention of the class was directed, was one of chronic inflammation of the conjunctiva in both eyes, with much thickening of the membrane, and a rough granular condition of its surface; and considerable opacity of the delicate layer reflected over the surface of the cornea.

This case was the sequel of an acute attack of conjunctivitis taking place more than six months previous. The conjunctiva lining the upper lids was very thick, hard and rough, and by its contact with the cornea contributed much to produce and perpetuate the superficial opacity which now almost destroys the vision. There is still a morbid sensitiveness of the eyes, as indicated by the free flow of tears when they are opened for examination.

*Treatment.*—The upper lids were everted and a smooth stick of nitrate of silver applied, so as to thoroughly cauterize the granular surface. The coagula were washed away with a camel hair pencil, and the lids allowed to close. It was remarked that this application should be repeated about every third day, until the granulations were removed. In the interval between these applications, a solution of sulph. morphine 5 grs. to the ounce of water, may be dropped into the eyes three or four times a day to lessen their irritability. This patient had been much debilitated by the protracted use of cathartics and low diet, and he was ordered a teaspoonful of the tincture of cinchonæ three times a day, each holding in solution the sixteenth of a grain of bi-chloride of mercury.

